# Enhanced cattle identification using Siamese network and MobileViT with EMA attention

**DOI:** 10.3389/fvets.2025.1660163

**Published:** 2026-01-21

**Authors:** Mingshuo Han, Baoshan Li, Qi Li, Yueming Wang, Mei Yang, Chang Gao

**Affiliations:** 1 School of Digital and Intelligence Industry, Inner Mongolia University of Science and Technology, Baotou, China; 2 School of Automation and Electrical Engineering, Inner Mongolia University of Science and Technology, Baotou, China; 3 Engineering Training Center of Inner Mongolia University of Science and Technology, Baotou, China

**Keywords:** attention module, cattle identification, deep learning, MobileViT, Siamese network

## Abstract

Accurate identification of individual cattle is paramount in livestock insurance to combat fraud. However, the performance of existing muzzle recognition methods degrades in complex scenarios involving occlusion or multi-angle views. This study addresses this limitation by first constructing a comprehensive cattle muzzle image dataset encompassing frontal, multi-angle, and occluded conditions. We then propose CattleMuzzleNet, a lightweight recognition model that integrates a siamese network, an enhanced MobileViT backbone, and an Efficient Multi-scale Attention (EMA) mechanism for robust feature extraction and matching. Its efficacy is systematically validated through comparative experiments on feature extraction networks, ablation studies on the attention mechanism, and confidence threshold analysis. Evaluated on a dataset of 31,312 images from 658 cattle, CattleMuzzleNet achieved an accuracy of 97.87% and an F1-score of 98.89%, with a compact model size of 6.9 MB. The results demonstrate high accuracy and robustness in complex scenarios, providing an effective technical solution for identity verification in cattle insurance.

## 1 Introduction

Accurate cattle identification is imperative for the verification of insurance claims (1), yet conventional methods are encumbered by substantial limitations. Permanent techniques, such as ear notching, branding, and microchip implantation, have been observed to cause animal discomfort and are subject to degradation over time (2, 3). Semi-permanent methods (e.g., ear tags, ID collars) are vulnerable to fraud (4, 5), while temporary solutions such as RFID technology impose high costs and practical constraints (6). These challenges underscore the pressing need for more reliable identification systems.

The advent of information technology has enabled the development of non-contact identification techniques that leverage biometric characteristics. These techniques have garnered significant attention due to their distinctiveness, permanence, cost-effectiveness, operational simplicity, and enhanced animal welfare (7). These approaches have emerged as a potential trend in cattle identification. Biometric-based non-contact identification methods (8), including facial patterns (9–12), retinal vasculature (13–15), and iris characteristics (16), are achieved through automated, vision-based recognition. However, post-mortem identification remains problematic. Iris recognition becomes infeasible, and facial recognition is hindered by rigor mortis. Additionally, the dark pigmentation of cattle coats can impede the perceptual discernment of coat pattern characteristics (17). Conversely, cattle muzzle patterns offer (49) high-contrast textures that maintain structural integrity after death, providing a distinctive and dependable biometric solution for insurance-related identification scenarios. Consequently, the employment of cattle muzzle print recognition technology exemplifies a pioneering approach to the identification of cattle. The technology’s distinctive biometric capabilities enhance the accuracy and reliability of insurance operations, thereby propelling the modernization of livestock insurance.

In preliminary studies, researchers primarily employed traditional computer vision algorithms to process cattle muzzle print images for feature extraction and identification. Kumar, S (18) proposed an automated recognition method based on a multiple linear regression framework, solved using group sparse signal representation and L2 minimization. This method achieved an identification accuracy of 93.87% on a cattle muzzle image database, outperforming other machine learning approaches. Ali Ismail Awad et al. (19) pioneered the use of the Bag of Visual Words (BoVW) model for cattle recognition, extracting features using SURF and MSER detectors, with SURF achieving the highest accuracy of 93%. Worapan Kusakunniran et al. (20) introduced a method that first locates the muzzle region using a Haar cascade classifier, then extracts keypoints via SIFT to build a bag of words, and finally classifies using an SVM, attaining 90% accuracy across different datasets. Amanpreet Kaur et al. (21) utilized the Shi-Tomasi corner detector combined with SIFT and SURF descriptors for feature extraction, along with linear dimensionality reduction to speed up computation. On a dataset of 930 cattle, the recognition accuracies of multilayer perceptron, decision tree, and random forest were 69.32, 74.88, and 79.60%, respectively, which were further improved to 83.35% through ensemble methods.

Given their limitations with complex scenes, fine-grained details, and computational efficiency at scale, traditional methods have ceded primacy to deep learning. CNNs, with their capacity for automatic feature learning, now deliver far greater speed and accuracy in cattle muzzle recognition. Santosh Kumar et al. (22) proposed a method combining CNNs, deep belief networks (DBNs), and stacked denoising autoencoders, achieving a rank-1 recognition accuracy of 98.99% on a cattle muzzle image database. Similarly, BELLO, R.-W (23) utilized stacked denoising autoencoders and DBNs for texture feature extraction, also attaining 98.99% accuracy, validating their efficacy in animal biometrics. Shojaeipour et al. (24) designed a two-phase YOLOv3-ResNet50 approach, employing few-shot learning to reduce data requirements, and achieved 99.13% accuracy in muzzle detection and 99.11% in biometric recognition. Guoming Li et al. (25) evaluated 59 deep learning models on a dataset of 4,923 muzzle images, with the best model reaching 98.7% accuracy and a processing speed of 28.3 ms per image. Santosh Kumar (26) developed a health monitoring system integrating behavioral data and muzzle prints, using SVM and incremental decision tree classifiers to achieve 97.99% identification accuracy. G. N. Kimani et al. (27) applied Wide ResNet50 and VGG16BN models to a dataset of 4,923 muzzle images from 268 calves; after image compression, ResNet50 reached 99.5% accuracy. Lee et al. (28) employed YOLOv8 and EfficientNet v2 on images of Korean cattle, with the best model achieving a validation accuracy of 0.981. To address occlusion in intensive farming, D. Li et al. (29) proposed a multi-feature decision-layer fusion method combining face, muzzle print, and ear tag features, attaining 95.74% overall accuracy (93.94% for muzzle prints). Pathak et al. (30) introduced an attention-based multimodal fusion technique, with muzzle-only recognition at 99.47% accuracy, increasing to 99.64% when combined with facial features. Russel et al. (31) developed a CNN method incorporating Gabor filters, achieving 98.99% identification accuracy on a beef cattle muzzle print database. Bara et al. (32) created a lightweight system based on SqueezeNet for Vrindavani crossbred cattle, which reached 97.22% accuracy while maintaining a model size under 4 MB.

The study’s key innovations are as follows:

1. Construction of a snout pattern dataset encompassing diverse angles and occlusion scenarios, comprising 31,312 images from 658 cattle, providing robust data support for accurate cattle identification. The dissemination of this muzzle pattern dataset is anticipated to serve as a pivotal resource within the scientific community, thereby enhancing the reproducibility and repeatability of research endeavors.
2. In addressing the necessity for precise cattle identification in the context of livestock insurance, with particular reference to the challenges of muzzle pattern recognition under multi-angle variations and occlusions, this paper proposes CattleMuzzleNet: a muzzle pattern recognition model based on a Siamese neural network architecture. The model has been built upon the MobileViT network and incorporates an EMA multi-scale attention mechanism (50). The aim of this model is to enhance recognition accuracy, particularly in addressing perspective variations and occlusion issues, thereby improving practical application precision.
3. The CattleMuzzleNet model has been developed to ensure high recognition accuracy while meeting lightweight standards (33). This facilitates convenient mobile device usage in real-world cattle insurance applications while ensuring on-site claims processing precision.

## 2 Materials and methods

### 2.1 Technical approach

**Figure 1:**
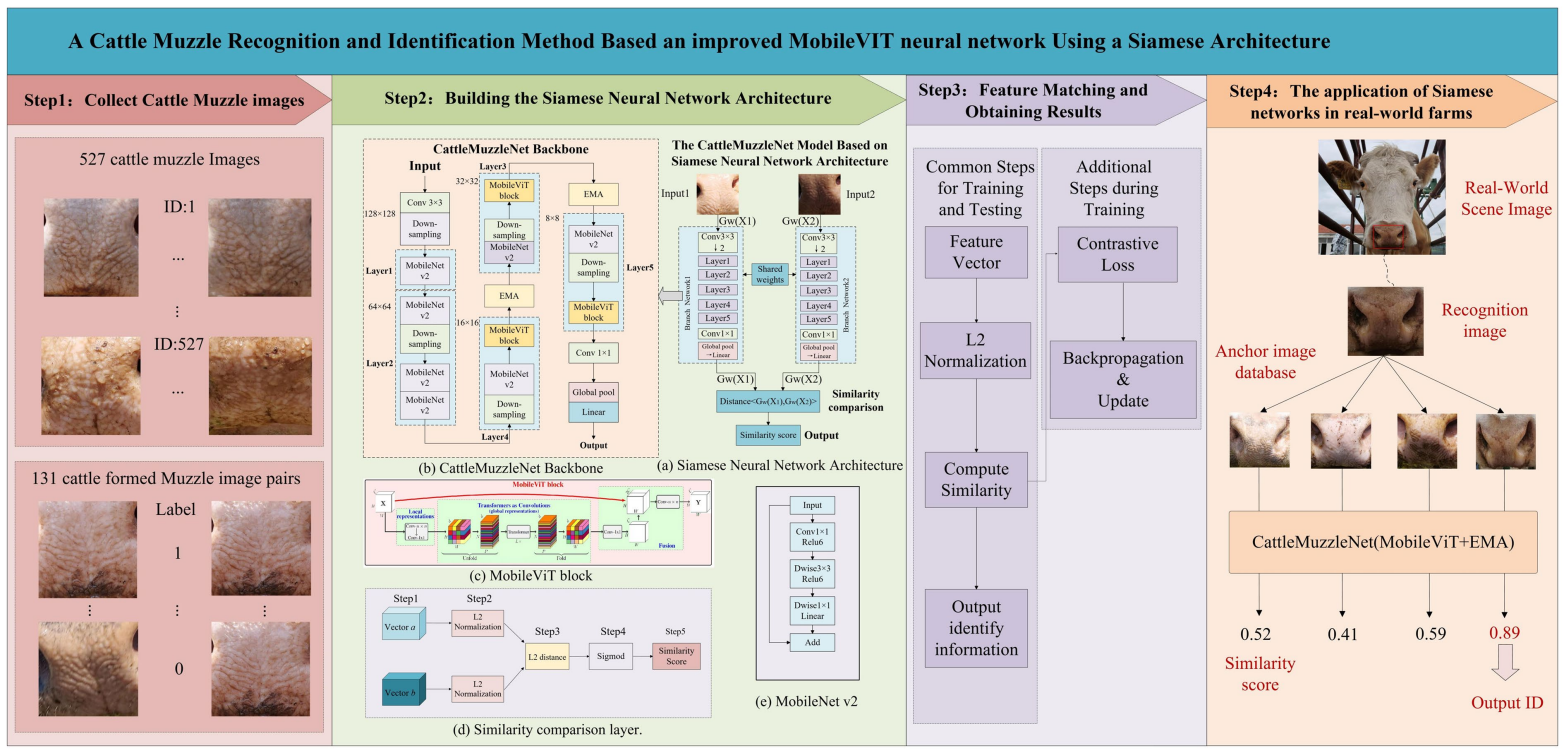
Technical approach for cattle muzzle pattern recognition task.

The technical approach for identifying cattle by their muzzle patterns is illustrated in Figure 1. The research process is methodically structured into four primary phases: data collection, construction of a Siamese neural network architecture, feature matching and result extraction, and application of the Siamese network in a real pasture.

First, during the data collection stage, we obtained a variety of cattle muzzle images from multiple farms and milk stations in the Inner Mongolia Autonomous Region, ensuring the collection of diverse and complex data. The data set under consideration encompasses images captured from multiple angles, with and without obstructions such as grass and feed. In the final analysis, a data set comprising 31,312 images from 658 cattle was constructed. To boost the diversity of the training data, we utilized data augmentation techniques such as random cropping, rotation, scaling, and illumination correction. This approach effectively augmented the robustness of the model.

In the subsequent stage of constructing the Siamese neural network architecture, we have suggested a Siamese neural network architecture incorporating a multi-scale attention mechanism, termed CattleMuzzleNet. This model employs a dual network configuration that utilizes shared weights to extract features from paired input images and subsequently calculates their similarity (34). To improve the model’s efficacy, a combination of MobileNetV2, MobileViT, and EMA (Efficient Multi-Scale Attention) modules was employed to effectively interpret photos of cattle muzzles from diverse perspectives.

During the feature matching and result extraction stage, the trained model extracts the image’s feature vector and performs L2 normalization to calculate the Euclidean distance between images and evaluate their matching. Training with a contrastive loss function (Contrastive Loss) (35) enables the model to accurately distinguish between matched and unmatched images, thereby improving the identification accuracy and robustness.

In the concluding phase of the project, the Siamese network was implemented on an actual farm. CattleMuzzleNet was implemented in an actual farm environment. The target detection model identifies the area of the cattle’s muzzle, and the camera captures the image. Subsequently, the image is compared with the images contained within the database, and a similarity score is generated. The most relevant image and the corresponding cattle identification (ID) are output, achieving efficient and accurate real-time identification. This technology offers innovative solutions for image recognition in the domains of cattle insurance and identity verification.

#### 2.1.1 Collection of data

**Figure 2:**
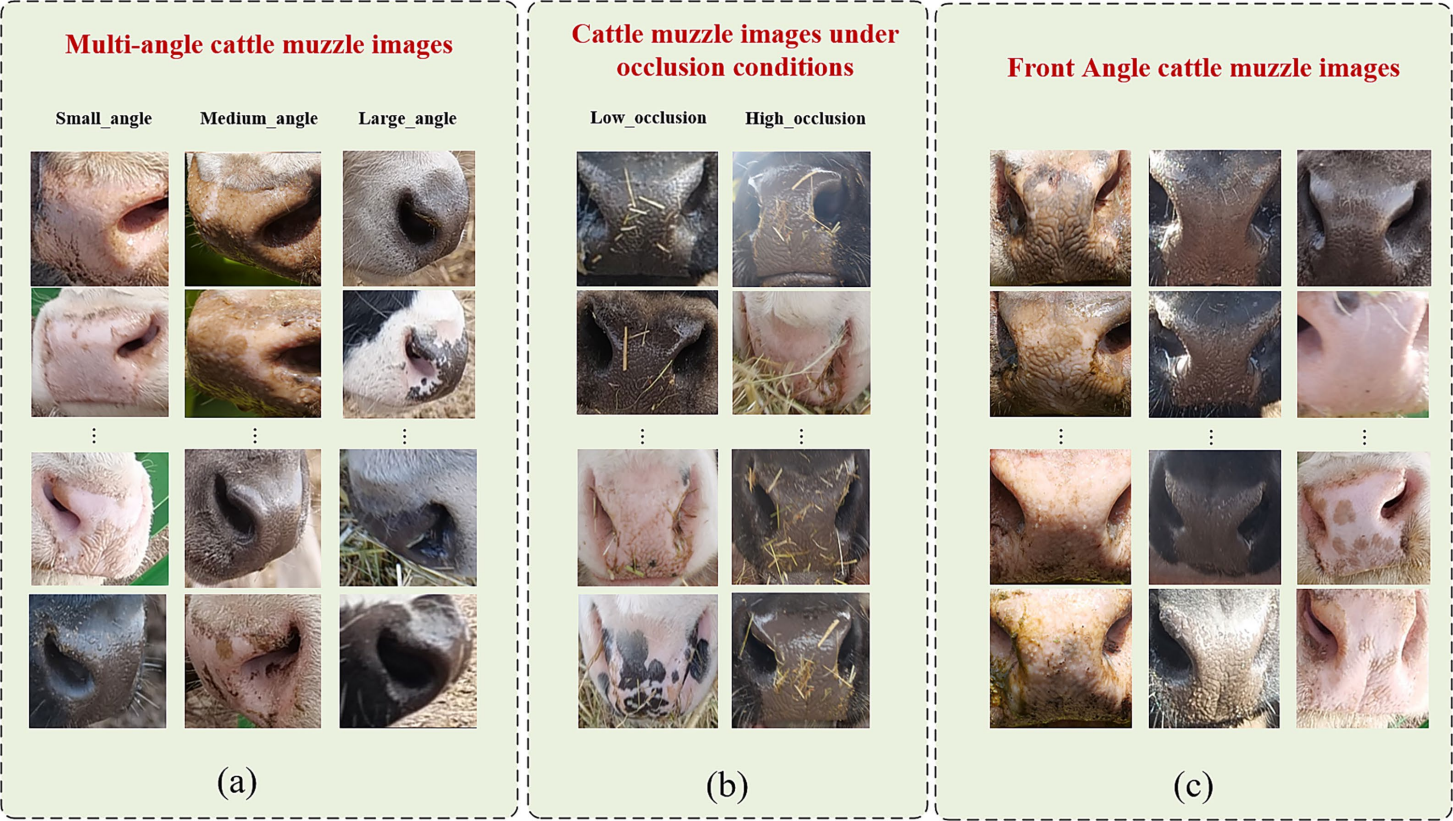
The cattle muzzle images. **(a)** shows multi-angle cattle muzzle images, **(b)** shows images under occlusion conditions, and **(c)** shows frontal angle muzzle images.

As demonstrated in Figure 2, the experimental data were collected from multiple pastures across Inner Mongolia using Panasonic GH5s high-definition cameras under clear weather conditions. The resulting dataset comprises 31,312 muzzle pattern images from 658 cattle (255 Wagyu, 329 Simmental, 74 Holstein), with each individual represented by 30–120 images covering frontal, occluded, and multi-angle views.

Images were manually cropped and annotated to highlight the muzzle region, then enhanced through data augmentation techniques including random cropping, rotation, scaling, and lighting modifications (36). Multi-angle images were categorized as Small (<30°), Medium (30–60°), or Large (>60°), while occluded images were classified as Low (<40% area) or High occlusion (>40%). These steps significantly improved the dataset’s diversity and realism, bolstering model robustness and generalization capacity. Representative samples are shown in Figure 2.

In the data collection and segmentation phase, the image dataset was partitioned into training and testing sets using stratified sampling (37) to maintain the equilibrium of data distribution and prevent breed-specific overfitting or underfitting. The dataset was divided in an 8:2 ratio, yielding 24,976 training images (527 cattle) and 6,336 testing images (131 cattle), with each breed proportionally represented across subsets. During evaluation, 6,000 image pairs—labeled as “match” (1) or “mismatch” (0) —were constructed from the test set to quantitatively assess the model’s recognition capability and robustness across diverse image types.

#### 2.1.2 Building a Siamese neural network architecture

This paper introduces CattleMuzzleNet to address key challenges in cattle muzzle pattern recognition for insurance applications, such as feed obstruction and multi-angle variations which impair recognition accuracy and claims processing efficacy. As outlined in Figure 1, Step 2, the model employs a Siamese neural network architecture integrated with a multi-scale attention mechanism (38) to tackle these issues. This model’s core principle is to extract features from a pair of input images utilizing two sub-networks with shared weights, followed by a comparison of their similarity. The network is predominantly comprised of two identical sub-network branches, with each branch responsible for extracting feature information from the input images and evaluating the relationship between images by calculating similarity. In the Siamese neural network, the inputs are two images, X1 and X2, which are processed through a neural network W with shared weights. The network maps the two input images to a new feature space, represented as GW (X1) and GW (X2). Subsequently, the network calculates the similarity between the two inputs using a loss function (Loss). Throughout the training process, the network is presented with a pair of images alongside their respective labels, which are defined by the categories of the input images. If the two images are from the same category, the label is assigned a value of 1; if they are from different categories, the label is assigned a value of 0. The Siamese neural network learns to consolidate the feature vectors of analogous images and differentiate the feature vectors of disparate images by evaluating the similarity between pairs of images. The distinctive architecture of the Siamese neural network is defined by its capacity to process paired sample data, which can be constituted by two images belonging to the same category or two images from different categories. This configuration effectively increases the number of input samples for the network, thereby enhancing its capacity for training. The flexibility of this input method leads to an augmentation in the diversity of training samples, which, in turn, contributes to the enhancement of the model’s generalization ability. A comparison of traditional cattle muzzle print recognition methods with those of Siamese neural networks reveals significant advantages in feature extraction methods, similarity measurement, and the training process. The utilization of metric learning facilitates the enhancement of neural networks’ capacity to effectively process the similarity between images, thereby improving the precision and resilience of cattle muzzle print recognition.

The CattleMuzzleNet framework integrates a feature extraction network and a similarity comparison layer, leveraging a lightweight Transformer-based design that synergizes convolutional neural networks’ local feature extraction capabilities with the global contextual awareness of self-attention mechanisms (39). Optimized for mobile deployment, the model employs a multi-component architecture comprising standard convolutional layers, MobileNetV2 and MobileViT modules, an EMA attention block, global pooling, and a fully connected layer (40, 41) to achieve efficient and accurate recognition.

The feature extraction process commences with standard convolution, progressing through cascaded MobileNetV2, EMA, and MobileViT modules for hierarchical processing. MobileNetV2 employs inverted residual blocks with depthwise separable convolutions and linear bottlenecks to optimize representational power while preserving computational efficiency (42). MobileViT employs sequence modeling via spatial unfolding, Transformer-based encoding, and folding operations to capture long-range dependencies, enhanced by skip connections for localized feature preservation (43). This hybrid design ensures robust feature fusion, significantly improving discriminative accuracy and scalability in livestock identification tasks.

**Figure 3:**
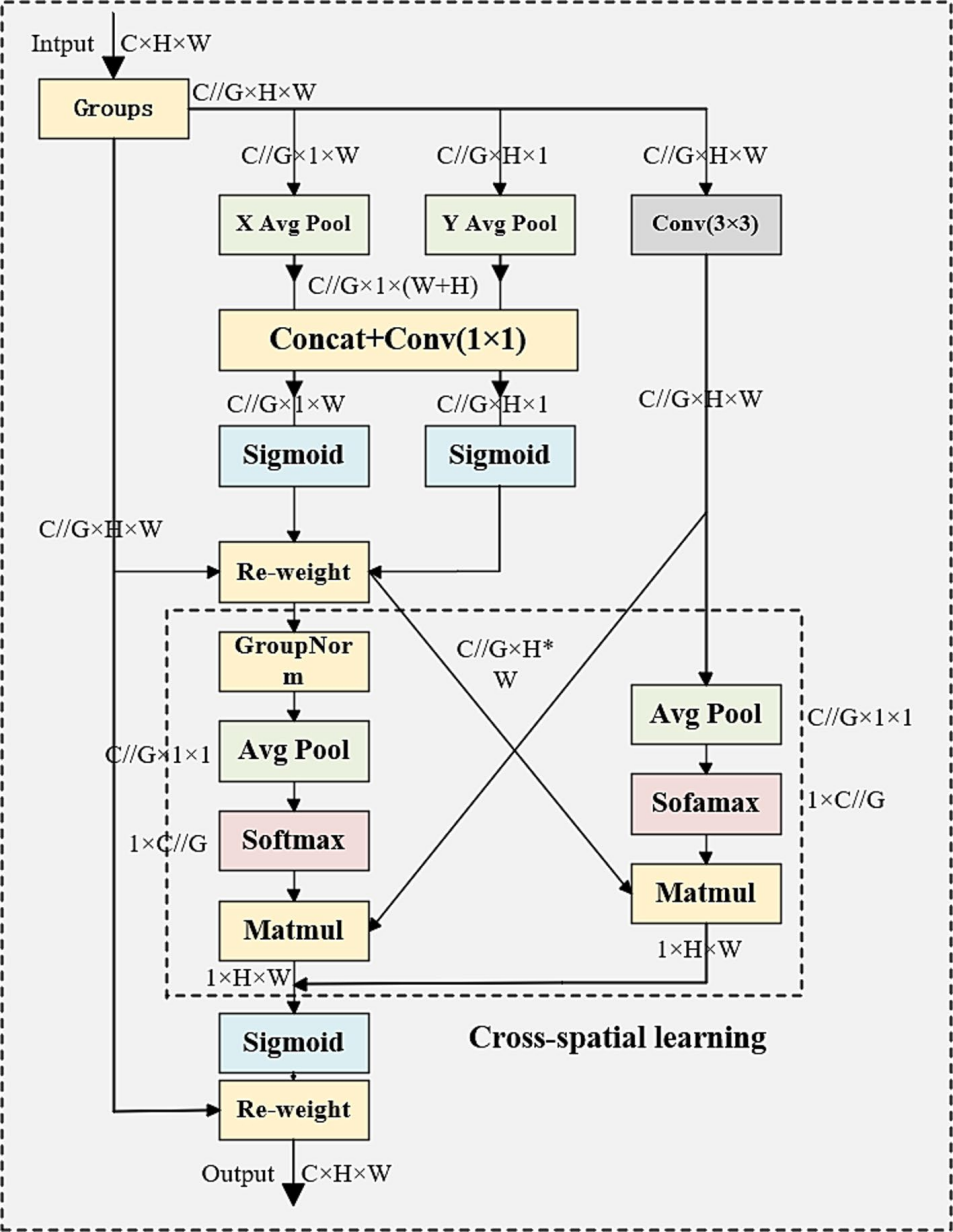
Illustration of EMA module. In this context, “G” denotes the segmented groups, “X Avg Pool” signifies the one-dimensional horizontal global pooling, and “Y Avg Pool” refers to the one-dimensional vertical global pooling, respectively.

To improve the model’s accuracy in identifying cattle muzzle prints, the Efficient Multi-Scale Attention (EMA) mechanism was integrated, as depicted in Figure 3. The EMA module utilizes an innovative, efficient, multi-scale parallel subnetwork architecture to preserve information across each channel while minimizing computational burden. This module readjusts the channel weights in each parallel branch by encoding global information and subsequently consolidates the output features of the two parallel branches through cross-dimensional interactions to capture pixel-level pair relationships.

The EMA module’s design is based on cross-dimensional information interaction and multi-scale feature extraction concepts. The EMA module employs two parallel branches: one utilizing 1×1 convolution to capture local features and the other employing 3×3 convolution to capture global features. The integration of these two branches enables the EMA module to efficiently process cross-channel and cross-spatial dependencies without compromising the channel dimension. The integration of features across various scales is a critical aspect of EMA, as it facilitates the capture of multi-scale information with greater efficiency, thereby mitigating information loss that can occur with dimension reduction. This module employs a fusion of attention maps from parallel subnetworks through cross-spatial learning methods, with the objective of accentuating global contextual information. This approach enables the attainment of enhanced efficiency while maintaining high performance. The EMA module has demonstrated its ability to optimize computational resource usage while preserving a high level of recognition accuracy. It has also exhibited the capacity to adjust to discrepancies in cattle muzzle prints across various poses, thereby augmenting the network’s overall resilience and recognition proficiency.

The similarity comparison layer of the CattleMuzzleNet model, as depicted in Step 2 (d) is tasked with quantifying the similarity between two images of cattle muzzle patterns and translating this into a similarity score. The process commences with the extraction of feature vectors a and b from the two images using a feature extraction network. Thereafter, these feature vectors are subjected to L2 normalization to standardize their lengths and project them onto the unit sphere. The similarity between the two feature vectors is ultimately assessed using the Euclidean distance metric, as illustrated in Equation 1:


$$d=\sqrt{\sum_{i=1}^{n}{\left(a_{i}-b_{i}\right)}^{2}}$$ (1)

In this formula, *n* denotes the dimension of the feature vector, while ai and bi represent the values of vectors a and b in the i-th dimension, respectively. Subsequent to calculating the L2 distance, the Sigmoid function is employed to map the distance values to similarity scores between 0 and 1. The output range of the Sigmoid function is [0, 1], representing the probability value of similarity. Equation 2 defines the sigmoid function:


$$\text{Sigmod}(x)=\frac{1}{1+e^{-x}}$$ (2)

In this particular instance, x denotes the calculated Euclidean distance. In this manner, smaller Euclidean distances (i.e., image features are highly similar) will receive similarity scores that approximate 1, while larger Euclidean distances (i.e., image features are highly dissimilar) will receive similarity scores that approximate 0. The network ultimately produces a similarity score that quantifies the resemblance between the two images. A score nearing 1 signifies a substantial similarity between the images, whereas a score nearing 0 denotes minimal similarity.

Innovative design has been utilized to improve the precision and reliability of cattle muzzle pattern recognition. While concurrently ensuring that computational complexity remains at a minimal level. This augmentation in accuracy and robustness is achieved by CattleMuzzleNet. The network design encompasses the incorporation of an EMA module into the initial and secondary MobileViT modules. Following the implementation of two introductory sessions regarding the EMA module, the model’s capacity to represent image features has undergone a substantial enhancement. Consequently, CattleMuzzleNet is now capable of more accurately capturing both local and global image details, thereby enhancing recognition accuracy and model robustness.

#### 2.1.3 Feature matching and result extraction

As illustrated in Figure 1, the third step involves the process of feature matching and result extraction, feature matching plays a fundamental role in deep learning (44). In this step, the efficacy of the trained model is assessed by evaluating its performance on a set of images designated for testing purposes. Initially, the model extracts the feature vectors of the images. These feature vectors reflect the unique details of each image and represent the key features of the cattle’s muzzle. Subsequently, L2 normalization is performed on the extracted feature vectors. The objective of L2 normalization is to standardize the length of the feature vectors so that they are mapped to a unit sphere. This process ensures more accurate and stable calculations of the similarity between feature vectors. Specifically, L2 normalization is achieved by dividing the feature vectors by their L2 norm, as demonstrated in Equation 3. This process ensures that similar images are proximate in the feature space, while dissimilar images are comparatively distant from each other.


$$\text{normalized}\_\text{features}=\frac{\text{features}}{{\left\|\text{features}\right\|}_{2}}$$ (3)

Subsequent to the extraction and normalization of feature vectors, the Siamese network utilizes this data to determine the category to which two images belong by calculating their similarity. Specifically, the Euclidean distance is employed to calculate the similarity, and subsequently, a threshold is established to determine whether the similarity of the two images exceeds the threshold. In the event that the similarity exceeds the established threshold, the recognition is deemed successful.

During the training of the model, a contrastive loss function was specifically employed for the purpose of optimization. Equation 4 illustrates the equation for the contrastive loss function.


$$\text{Loss}=\frac{1}{N}\sum_{i=1}^{N}y_{i}\times d^{2}+(1-y_{i})\times \max{\left(0,m-d\right)}^{2}$$ (4)

Among them, *N* signifies the number of sample pairs, y_i_ denotes the label of the sample pair (if the image pair belongs to the same class, then y_i_ = 1, otherwise 0), d signifies the Euclidean distance between the image pairs, and m signifies a fixed threshold.

The contrastive loss function plays a critical role in enhancing feature discrimination by minimizing inter-class distances while maximizing intra-class separation within the embedding space. Through backpropagation-guided optimization, this approach enables the Siamese network to learn semantically rich representations of cattle muzzle patterns, progressively improving recognition accuracy through iterative weight updates.

While traditional methods (e.g., Softmax Loss and Triplet Loss) struggle with open-set scenarios—Softmax incurring parametric explosion with identity increments, and Triplet Loss requiring computationally intensive hard-negative mining—Siamese networks circumvent these issues by direct pairwise similarity computation. This architecture ensures efficient training without scalability bottlenecks, maintains robust accuracy on unseen identities, and optimizes feature embedding cohesion through contrastive learning. By clustering analogous patterns while distancing dissimilar features, the framework significantly enhances precision and robustness in cattle muzzle recognition, establishing a superior paradigm for open-set biometric applications.

#### 2.1.4 Application of Siamese neural networks in practical scenarios

As illustrated in Figure 1, Step 4 delineates the comprehensive process of the CattleMuzzleNet model, which is predicated on a Siamese neural network architecture for the identification of cattle muzzle patterns in a real pasture. In the actual identification process, an image of each muzzle in the pasture is stored as an anchor image. Upon initiation of the identification process, the camera captures images of the muzzles from the actual pasture scene and identifies the muzzle pattern area through an object detection model. Subsequently, the captured muzzle pattern images are compared one by one with the anchor images of each muzzle in the server through the CattleMuzzleNet model. The process of comparison generates a similarity score based on the Euclidean distance between the two feature vectors, thereby indicating the degree of similarity between the input image and each image in the database. The score’s magnitude reflects the extent of similarity between the two images. By analyzing the similarity scores of all images, the system determines the image exhibiting the greatest similarity to the input image and provides the associated identification code. This process provides a recognition result, indicating the object or category to which the input image belongs.

## 3 Results

All experiments in this study were performed under the specified hardware conditions: The device features an Intel i9 9900K processor with a frequency of 3.60 GHz, two NVIDIA GeForce RTX 2080 Ti GPU with 12 GB of memory, and 64 GB of RAM. The operating system is Ubuntu 18.04, equipped with CUDA 10.0, cuDNN 7, PyTorch 1.7, and Python 3.6.9. During the model’s training, the input image dimensions were established at 105 × 105 × 3. The initial learning rate was established at 0.001 and progressively diminished to 0.0001. The training rounds were established at 200, the batch size at 32, and the optimizer at Adam.

### 3.1 General framework

This study employs accuracy, precision, recall, and F1-score to evaluate the classification performance of the CattleMuzzleNet network on the muzzle print image dataset. Furthermore, in order to evaluate the model’s performance in challenging scenarios in a more comprehensive manner, particularly with regard to the requirements of real-world cattle identification applications, Top-K accuracy is introduced as a crucial supplementary evaluation metric (45). The subsequent formulas have been formulated for each metric:

Accuracy refers to the ratio of correctly identified samples to the entire amount of test samples. The calculation formula is presented in Equation 5:


$$\text{Accuracy}=\frac{\mathrm{TP}+\mathrm{TN}}{\mathrm{TP}+\mathrm{TN}+\mathrm{FP}+\mathrm{FN}}\times 100$$ (5)

Among these, TP (True Positives) refers to true examples, TN (True Negatives) refers to true negative examples, FP (False Positives) denotes instances of false positives, while FN (False Negatives) signifies instances of false negatives.

The rate of accuracy is the ratio of correctly identified as positive class examples to the total number of samples predicted as positive class. The calculation formula is presented in Equation 6.


$$\text{Precision}=\frac{\mathrm{TP}}{\mathrm{TP}+\mathrm{FP}}\times 100$$ (6)

The recall rate is the proportion of accurately identified positive samples to the total number of positive samples. The calculation formula is presented in Equation 7.


$$\text{Recall}=\frac{\mathrm{TP}}{\mathrm{TP}+\mathrm{FN}}\times 100$$ (7)

The F1-Score is the harmonic mean of precision and recall, utilized to thoroughly represent the overall efficacy of a model. The calculation formula is presented in Equation 8.


$$F1-\text{Score}=\frac{2\times \text{Precision}\times \text{Recall}}{\text{Precision}+\text{Recall}}\times 100$$ (8)

Top-K Accuracy is a pivotal metric in retrieval tasks, employed to evaluate model performance in large-scale individual identification (1:N retrieval) scenarios. This figure is indicative of the proportion of correct labels appearing among the top K results with the highest predicted probabilities, the calculation formula is presented in Equation 9:


$$\mathrm{Top}-K\;\text{Accuracy}=\frac{N_{\text{correct}@K}}{N_{\text{total}}}\times 100$$ (9)

It is customary to assign integer values to K, such as 1, 5, or 10. Top-1 accuracy requires the model to rank the correct individual first, while Top-K accuracy (K > 1) evaluates the model’s ability to include the correct individual within the candidate set when precise matching is unattainable. For instance, an enhancement in Top-10 accuracy signifies the model’s augmented capacity to facilitate human review by offering a more refined set of candidates, consequently enhancing system efficiency.

### 3.2 Comparative experiments of feature extraction networks

In the domain of cattle insurance, the objective was to ascertain an equilibrium between the precision of image recognition and the computational efficiency of positive muzzle pattern images, multi-angle images, and occluded images. To this end, a series of comparative experiments were conducted on several classic deep learning models, including the VGG series network, MobileNet series network, and MobileViT family. The primary objective was to select a baseline model that exhibited both high accuracy and minimal computational demands. All models were trained on a muzzle print dataset and assessed using a test dataset to verify the reliability and applicability of the experimental outcomes. During the testing phase, a dataset comprising over 6,000 image pairs was utilized, with each pair being labeled as either “match” (1) or “mismatch” (0), thereby enabling the assessment of the model’s recognition capability. The trained models extract image feature vectors and calculate the similarity between two images. The similarity between the images is determined by computing the Euclidean distance and utilizing the sigmoid function. Subsequently, a threshold is established to ascertain whether the images are congruent. In the event that the similarity exceeds the established threshold, the recognition is deemed successful. This process assists in evaluating the model’s performance and stability across various image types.

The experimental findings are displayed in Table 1, where the MobileViT-S model demonstrates superior performance across all key metrics, thus substantiating its preeminence in this study. Specifically, MobileViT-S exhibited exceptional accuracy (97.37%), precision (96.77%), recall (97.17%), and F1 score (97.40%), underscoring its remarkable capacity to discern muzzle patterns in intricate environments. This finding suggests that MobileViT-S not only accurately identifies the muzzle pattern features of cattle but also effectively handles complex conditions such as lighting variations, pose changes, and grass obstruction in farm environments, ensuring high-precision identification during the claims process. The MobileViT-S model has a size of 6.7 MB, slightly exceeding that of some lightweight models, such as SqueezeNet at 1.2 MB and MobileNetV2 at 4.2 MB, with SqueezeNet exemplifying a commendable equilibrium between accuracy and model dimensions. Consequently, MobileViT-S was designated as the reference model for this research.

**Table 1:** The evaluation of the baselines for different recognition models.

Baseline	Accuracy	Precision	Recall	F1-Score	Total parameters
VGG16	94.88	91.70	97.09	95.07	138
VGG16BN	95.74	94.98	96.59	95.78	138
VGG19	93.92	90.08	97.02	94.20	143
VGG19_BN	96.67	95.15	97.10	96.72	143
MobileNetV2	95.73	95.06	96.46	95.76	4.2
MobileNetV3	96.19	95.53	96.91	96.22	5.4
ResNet50	96.48	95.42	96.64	96.52	25.6
RegNet	97.32	96.40	97.11	97.35	39.2
EfficientNetV1	96.70	96.20	96.17	96.68	5.3
EfficientNetV2	91.23	85.87	97.09	91.84	7.8
DenseNet	96.44	95.25	96.77	96.49	8.0
SqueezeNet	93.99	91.04	96.58	94.20	1.2
InceptionV3	94.94	91.86	97.13	95.12	23.9
MobileViT-XXS	96.56	96.26	96.87	96.57	2.9
MobileViT-XS	96.65	96.38	96.94	96.66	3.4
MobileViT-S	**97.37**	**96.77**	**97.17**	**97.40**	6.7
MobileViTV2_050	95.98	94.89	96.19	96.03	3.8
MobileViTV2_075	96.57	96.03	96.16	96.59	5.0
MobileViTV2_100	96.57	96.29	96.87	96.58	6.7
MobileViTV2_125	96.46	96.27	95.60	96.43	8.3
MobileViTV2_150	96.30	96.69	95.89	96.28	9.9
MobileViTV2_175	96.91	96.11	96.77	96.93	11.5
MobileViTV2_200	97.13	96.16	97.16	97.16	13.1

The bold values indicate that the performance of this model is the best.

### 3.3 Ablation experiments introducing attention mechanisms

An attention mechanism was integrated into the MobileViT-S model to improve its ability to recognize muzzle patterns under varying angles and occlusion conditions. This study performed ablation experiments to validate the actual effect following the introduction of the attention module. The aim of the experiment is to enhance the efficacy of MobileViT-S in muzzle print recognition by incorporating the EMA (Efficient Multi-Scale Attention) module, specifically addressing multi-angle variations and occlusion challenges of muzzle prints in real-world pasture settings. The MobileViT-S model serves as the foundational framework, with the dataset and experimental methodologies mirroring those employed in the feature network comparison experiments. The EMA module is integrated subsequent to various network layers, resulting in the formulation of four distinct experimental models for comparative analysis. The first group consists of the unmodified MobileViT-S model, the second group is MobileViT-S + EMA-3 (with an EMA module added after the third layer), the third group is MobileViT-S + EMA-4 (with an EMA module added after the fourth layer), and the fourth group is CattleMuzzleNet (with an EMA module added after both the third and fourth layers).

**Figure 4:**
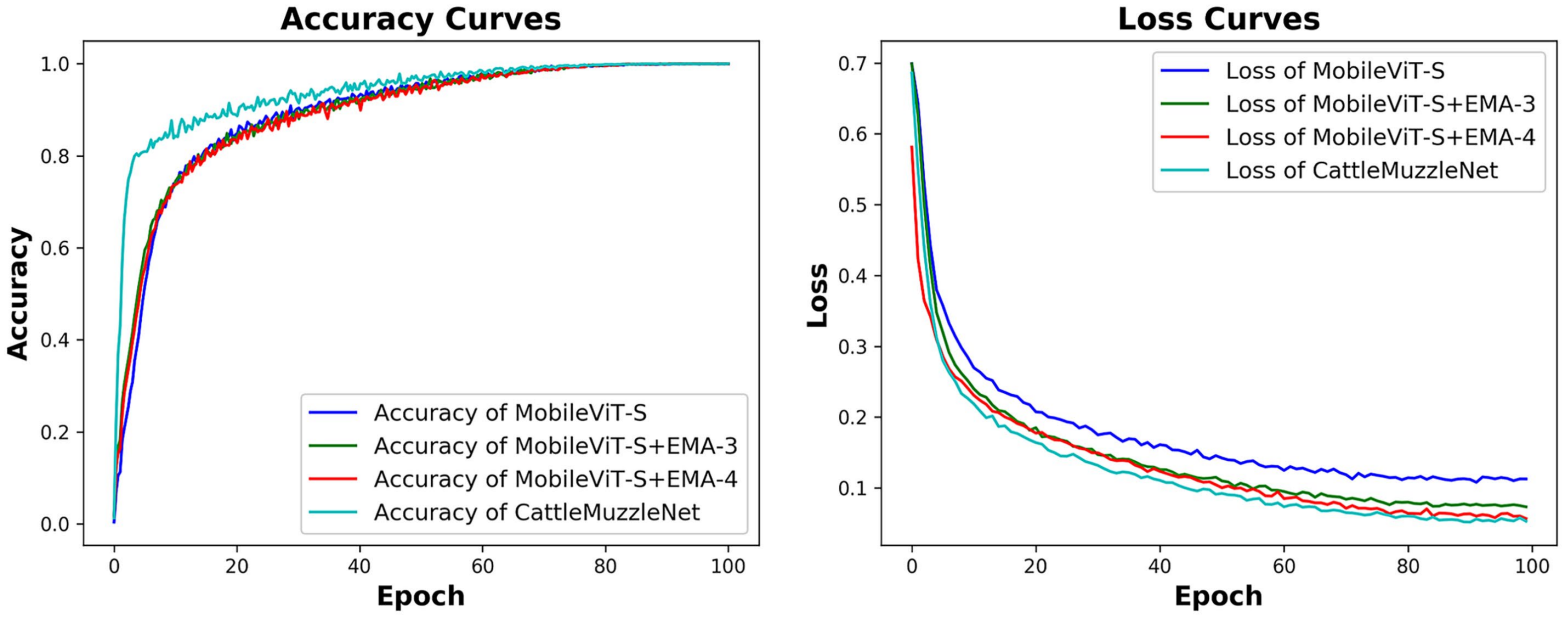
Comparative analysis of validation accuracy curves and training loss curves for models derived from ablation experiments on cattle muzzle images.

**Figure 5:**
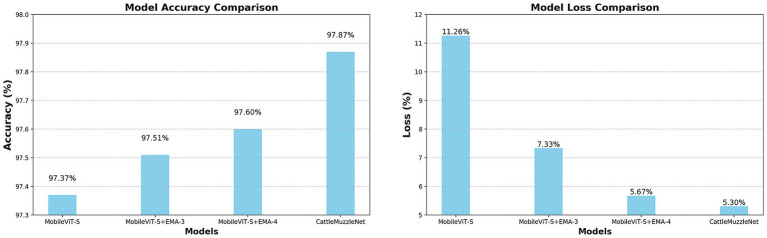
Accuracy and training loss in ablation experiments.

A comparative analysis of various models demonstrated that the incorporation of the EMA module resulted in a more stable convergence trend throughout the training of all enhanced models. As demonstrated in Figures 4, 5, the loss curves of MobileViT-S + EMA-3 and MobileViT-S + EMA-4 exhibited a substantial decrease compared to the baseline model, achieving lower training errors in a reduced number of training cycles. Concurrently, the accuracy curves demonstrate that the two models with the EMA module progressively exceed the baseline model during training, particularly with a notable enhancement in performance on the validation set. In conclusion, the incorporation of the EMA module within CattleMuzzleNet is instrumental in preserving the minimal loss value in the subsequent phases of training. Moreover, it facilitates a consistent enhancement in accuracy, thereby underscoring its proficiency in generalization and stability.

These experimental results validate the key role of the EMA module in improving network performance and stability, especially when facing multi-angle variations and occlusion issues in muzzle pattern recognition. The EMA module has been demonstrated to enhance the model’s robustness to a considerable extent. Consequently, the integration of the EMA module is of paramount importance in enhancing recognition accuracy, accelerating convergence speed, and augmenting the model’s generalization capability.

The results from the previously mentioned experiments are displayed in Table 2. The experimental results demonstrate that the performance of the enhanced model was markedly improved following the incorporation of the EMA module as outlined in this experiment. Compared to MobileViT-S, the accuracy of the two enhanced models improved by 0.14 and 0.23%, respectively, while the F1 scores rose by 1.26 and 0.64%, respectively, after the incorporation of a single EMA module. The introduction of two sets of EMA modules in parallel led to a marked enhancement in the model’s performance. In conclusion, the CattleMuzzleNet model, as proposed, demonstrated the most optimal performance in the cattle muzzle pattern recognition task, attaining an accuracy rate of 97.86% and an F1 score of 98.89%. Additionally, it exhibited the highest precision and recall metrics. Consequently, it can be inferred that the integration of two sets of EMA modules led to a minimal increase of 0.2 MB in model size, thereby illustrating that CattleMuzzleNet effectively achieves an optimal equilibrium between model size and recognition performance. This optimization facilitates the CattleMuzzleNet model’s capacity for efficient and accurate feature extraction in practical applications for cattle insurance, thereby significantly enhancing the fairness and efficiency of claims processing.

**Table 2:** Summary of the ablation experiment results.

Baseline	Accuracy	Precision	Recall	F1-score	Total parameters
MobileViT-S	97.37	96.77	97.17	97.40	6.7
MobileViT-S + EMA-3	97.51	99.77	97.58	98.66	6.8
MobileViT-S + EMA-4	97.60	98.18	97.89	98.04	6.8
CattleMuzzleNet	**97.87**	**99.84**	**97.96**	**98.89**	6.9

The bold values indicate that the performance of this model is the best.

In order to gain a more profound understanding of the accuracy (97.87%) of the optimal model (CattleMuzzleNet) in this study, a comparison was made with the accuracy of other models in the literature. As demonstrated in Table 3, the CattleMuzzleNet model exhibits a higher level of accuracy in comparison to the models proposed by D. Li et al. (29) and Bara et al. (32), yet falls below the methods introduced by Pathak et al. (30) and Russel et al. (31), with the maximum difference in accuracy being less than 2%. It is noteworthy that the dataset utilized in this study encompasses 31,312 images of 658 cattle, representing a substantially larger sample size in comparison to other studies. Furthermore, it encompasses a variety of complex scenarios, including multi-angle views and occlusions, thereby imposing higher demands on the model’s generalization capabilities. In the context of this more challenging dataset, CattleMuzzleNet attains performance that approaches optimal models while preserving a lightweight architecture, thereby demonstrating its strong applicability and comprehensive advantages in real-world farming environments.

**Table 3:** Comparison of the proposed model’s accuracy with other models proposed in the literature.

Target	Approach/model	Accuracy (%)	Dataset scale (cattle number, image number)	Reference
Muzzle	Siamese Network with EMA Attention for Lightweight Cattle Muzzle Identification	97.87	658 cattle, 31,312 images	Present research
Muzzle	Multi-feature decision-level fusion	93.94	194 cattle, 6,902 images	(29)
Muzzle	Attention-based multi-modal fusion	99.47	300 cattle, 2,900 images	(30)
Muzzle	Two-stream CNN with Gabor filters	98.99	268 cattle, 4,923 images	(31)
Muzzle	Lightweight SqueezeNet	97.22	264 cattle, 2,640 images	(32)

### 3.4 Model stability testing experiment

As demonstrated in Table 4, stability tests (46) were conducted on the CattleMuzzleNet model. Following the introduction of Gaussian noise and lighting variations, the model metrics demonstrated only minor declines (performance retention rate >97%), indicating a high degree of robustness against such pixel-level perturbations. This resilience is attributed to the EMA attention mechanism’s stable capture of key features. However, when confronted with geometric modifications (e.g., 30° rotation or 0.8x scaling), the model’s performance declined significantly (accuracy dropping to approximately 93%, with performance retention around 95%). This finding highlights a shortcoming in the model, namely its vulnerability to significant geometric transformations that are not adequately addressed in the training data. This is primarily due to the fact that the local structural relationships of the muzzle ridge texture features undergo changes during deformation, thereby posing challenges for feature matching. The findings from the experiment on compound interference suggest that the combined impact of multiple degradation factors can intensify the deterioration in performance. These findings suggest directions for future model optimization, such as introducing broader geometric transformations through data augmentation or incorporating feature descriptors insensitive to geometric deformations to enhance the model’s practicality in extreme real-world scenarios.

**Table 4:** Experimental results with different confidence thresholds.

Model	Confidence threshold	Accuracy	Performance retention rate
Baseline (original images)	–	97.87	100
Noise perturbation	Gaussian noise (σ = 0.05)	96.51	98.6
Illumination variation	Brightness decreased by 40%	95.33	97.4
	Brightness increased by 40%	95.89	98.0
Geometric modification	Clockwise rotation by 30°	93.47	95.5
	Scale changed to 0.8x	92.81	94.8
Compound perturbation	Brightness decreased by 20% + Noise (σ = 0.03)	94.25	96.3

### 3.5 Comparison of confidence threshold experiments for feature matching algorithms

In the feature matching process of muzzle ring pattern recognition, a confidence threshold is employed to ascertain the reliability of the matching results. In the context of employing a Siamese neural network for the purpose of feature matching, the determination of reliability in a match is contingent upon the comparison of two muzzle ring pattern images. Should the similarity score between these images exceed the predefined confidence threshold, the match is considered reliable. Conversely, if the similarity score falls below the predetermined threshold, the match is deemed unreliable, potentially due to the presence of noise or erroneous matching. The modification of the confidence threshold directly affects both the quantity and quality of the resulting matches. Consequently, the determination of a suitable confidence threshold is essential for the efficacy of the muzzle ring recognition task.

In this study, we evaluated the image pairs in the test set, where each pair of images may belong to the same muzzle (i.e., successful matching) or different muzzles (i.e., the experiment yielded unsatisfactory results when attempting to match the neural network Siameses). Nine unique confidence thresholds, spanning from 0.1 to 0.9 in increments of 0.1, were established, and the performance of the Siamese neural network was assessed at these differing confidence levels. The study primarily focused on the metrics of accuracy, precision, recall, and F1 score, emphasizing the changes observed in these metrics as the confidence thresholds were modified. Table 5 presents the experimental results, which demonstrate that a confidence threshold of 0.5 yields optimal matching performance, achieving an accuracy of 97.87%, precision of 99.84%, recall rate of 97.96%, and F1 score of 98.89%. However, although this threshold provides the optimal overall performance, it may result in false positives (i.e., Erroneous identification of negative samples as positive examples is particularly prevalent when the similarity between images is high). Consequently, in pragmatic implementations, establishing 0.5 as the threshold optimizes the matching quality while necessitating the management of the risk of false positives.

**Table 5:** Experimental results with varying confidence thresholds.

Model	Confidence threshold	Accuracy	Precision	Recall	F1-score
CattleMuzzleNet	0.1	83.91	84.74	83.55	84.27
	0.2	86.52	87.83	86.71	87.33
	0.3	90.13	92.35	89.36	91.44
	0.4	93.44	96.01	92.17	95.24
	0.5	**97.87**	**99.84**	**97.96**	**98.89**
	0.6	92.80	97.04	96.47	97.75
	0.7	89.34	91.32	88.47	91.74
	0.8	86.56	86.82	87.71	89.34
	0.9	84.41	85.52	86.54	85.12

The bold values indicate that the performance of this model is the best.

Conversely, when the confidence threshold is set at 0.9, the model demonstrates the least effective matching performance, with an accuracy rate of 84.41%, precision of 85.52%, recall rate of 86.54%, and F1 score of 85.12%. This outcome suggests that setting the confidence threshold excessively high results in the model’s failure to recognize some correctly matched samples, thereby leading to a reduction in recall. When determining an appropriate balance between accuracy and recall, a threshold of 0.5 is identified as the optimal choice for achieving optimal performance and matching quality in the task of identifying muzzle prints.

### 3.6 Experimental research on model visualization

**Figure 6:**
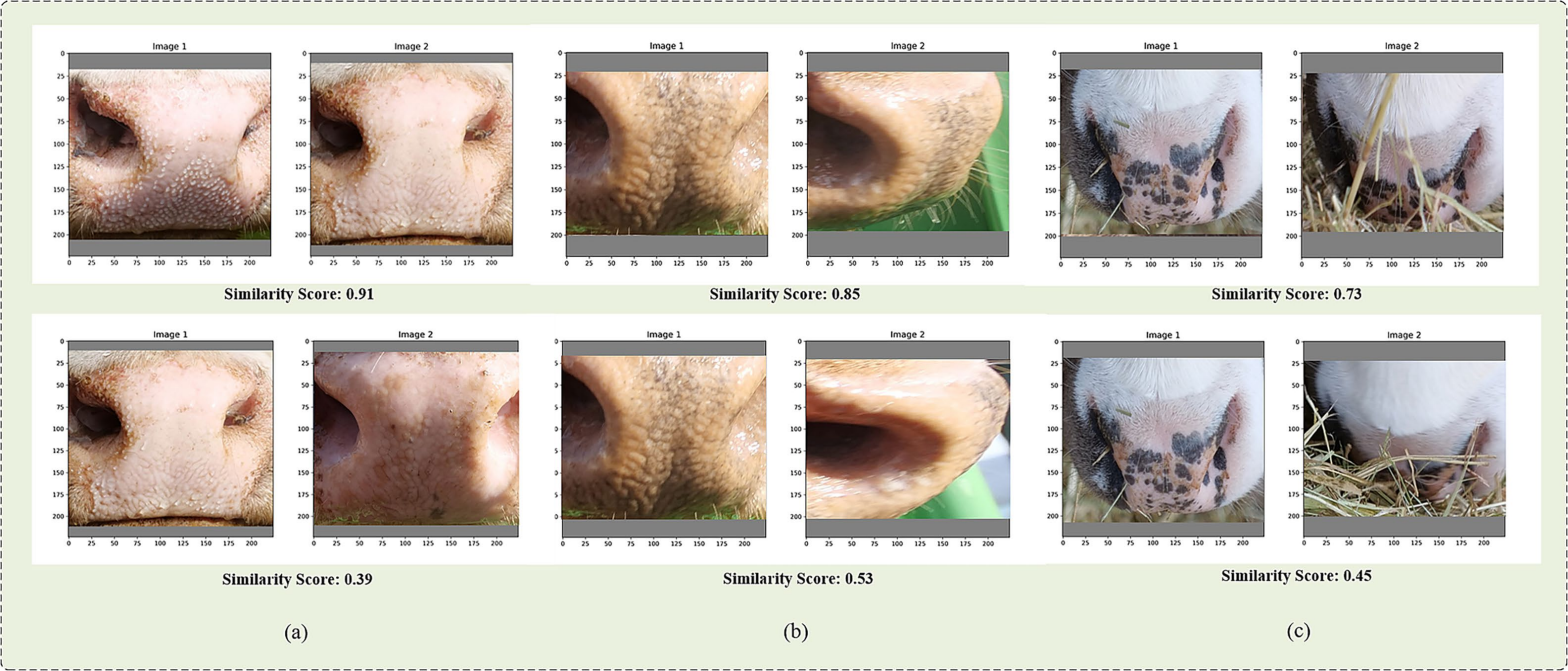
Cattle muzzle pattern distance and similarity score comparison. **(a)** shows the recognition results of frontal cattle muzzle prints, **(b)** shows the results from different angles, and **(c)** shows the results under occlusion conditions.

Figure 6 demonstrates the visual results of the suggested model for identifying muzzle patterns under various conditions are demonstrated. Initially, the two sets of images in Figure 6a present the model’s recognition results for frontal images, encompassing muzzle ring patterns of the same muzzle and those of different muzzles. The findings suggest that the model possesses the capacity to accurately differentiate between muzzle ring patterns of the same muzzle and those of different muzzles, thereby demonstrating its strong recognition capabilities. This performance advantage provides a reliable technical foundation for individual identity verification in the context of cattle farming insurance.

As illustrated in Figure 6b, two sets of images are presented, showcasing the model’s recognition performance at varying angles. The findings suggest that the model demonstrates optimal performance when the angle is approximately 45 degrees. However, as the angle approaches 60 degrees, the similarity score exhibits a notable decline, indicating that alterations in angle can substantially influence recognition accuracy. This observation warrants further investigation, as it potentially impacts the precision of insurance claims assessments.

As illustrated in Figure 6c, the recognition results are evident under conditions of grass obstruction. It has been observed that when the obstruction area is minimal, the model demonstrates an ability to accurately recognize the muzzle pattern. However, when the obstruction area is large, the recognition accuracy decreases. This indicates that the model’s recognition efficacy may be compromised in obstructed conditions, particularly in real breeding environments where impediments like grass or other obstacles are prevalent.

**Figure 7:**
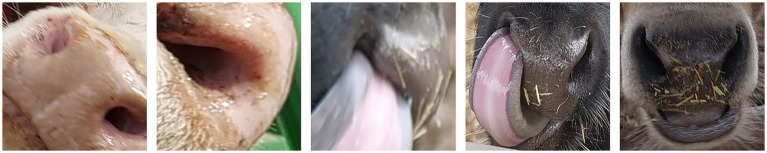
Cattle muzzle print images from failure cases.

In consideration of the aforementioned test results, it was determined that the CattleMuzzleNet model continues to demonstrate a proportion of misclassifications during the process of cattle muzzle pattern recognition. In order to further investigate the underlying mechanisms of model failure, a visual analysis of typical recognition failure cases was conducted, with representative examples shown in Figure 7. It is evident from the analysis that the accuracy of recognition is principally influenced by three factors. Firstly, when the cattle’s head is tilted beyond 60°, significant geometric distortion occurs in the muzzle pattern region, leading to increased feature matching errors. Secondly, when forage coverage exceeds 40%, local feature loss occurs, thereby weakening the model’s ability to infer texture topology. Furthermore, the presence of motion blur has been shown to have a detrimental effect on the clarity of the image, thereby disrupting the stability of feature extraction. These cases demonstrate the model’s perceptual limitations under extreme conditions. In order to address these issues, future research will focus on enhancing model robustness through two approaches: first, systematically expanding the dataset to include challenging samples such as large-angle head tilts and severe occlusions; second, introducing anti-interference training mechanisms to strengthen the model’s adaptability to incomplete or degraded features, thereby improving its practical performance in real-world scenarios.

To conduct an in-depth study of the model, we selected data from the test set to construct a dataset that does not contain frontal images but only includes multi-angle samples and occlusion samples. Subsequently, data augmentation techniques were employed, encompassing rotation, flipping, and brightness and contrast adjustments, on the image samples, culminating in a total of 9,960 images derived from 97 muzzles. The multi-angle samples were subsequently categorized into three distinct types: The angle is categorized as follows:

Small angle (less than 30 degrees, *n* = 1860).
Second item; Medium angle (30 degrees > angle > 60 degrees, *n* = 1920).
Large angle (angle > 60 degrees, *n* = 1980).

The occlusion samples are divided into three categories: The occlusion area was categorized as low (less than 40%, *n* = 2,100) or high (greater than 40%, *n* = 2,100), with the latter including scenarios such as feed adhesion and mud contamination.

As demonstrated in Table 6, within the medium angle range (Medium angle, 30°–60°), the model exhibits an accuracy rate of 88.32%. However, in the large angle (Large angle, >60°) scenario, the accuracy rate declines to 86.02%, accompanied by a 2.1 percentage point decrease in the recall rate. This performance degradation is attributable to alterations in head posture during cattle feeding and movement, thereby underscoring the necessity for the utilization of behavior guidance devices to optimize data collection angles during on-site inspections for insurance claims. In scenarios characterized by low occlusion, the model exhibits an accuracy rate of 90.76%. However, under high occlusion conditions, this performance metric declines to 86.41%. In particularly extreme cases, where muzzle pattern features are obscured by feed by more than 40%, the system’s reliance on the topological continuity of muzzle pattern edges for inference and recognition becomes pronounced. This phenomenon elucidates the rationale behind the recommendation, in open pasture environments, of the implementation of feeding management guidelines by farms, with the objective of reducing the probability of occlusion during data collection.

**Table 6:** Performance evaluation of classification model under multi-angle and occlusion conditions.

Dataset	Range	Count	Accuracy	Precision	F1-score
Multi-angle samples	Small angle	1,860	91.33	85.69	91.97
	Medium angle	1,920	88.32	81.29	85.00
	Large angle	1,980	86.02	70.57	78.78
Occluded_samples	Low occlusion	2,100	90.76	84.52	91.53
	High occlusion	2,100	86.41	77.15	87.10

This study’s experimental results highlight the limitations of the current research and provide valuable insights and guidance for future investigations. In the domain of cattle farming insurance, it is imperative to persist in the collection of datasets, augment the variety of cattle, and broaden the number of muzzle pattern samples under various angles and occlusion conditions. Furthermore, the model should be optimized to enhance its performance under large angles and significant occlusion, ensuring accurate identification of cattle information in various complex environments. This will contribute to the enhancement of risk management in the cattle farming insurance sector, leading to a reduction in fraud risks and the provision of more precise insurance services to farmers.

### 3.7 Large-scale cattle retrieval experiment

This study builds upon the completion of image pair verification (1:1 Verification) experiments, conducting further large-scale identity retrieval (1:N Identification) performance evaluations. This paradigm shift addresses practical requirements in real-world farming environments. While image pair verification is an effective means of assessing a model’s fundamental recognition capabilities, identity recognition tasks in actual livestock environments often require systems to rapidly and accurately retrieve target objects from extensive individual databases. Consequently, large-scale retrieval experiments offer a more comprehensive evaluation of a model’s practicality and robustness in complex application scenarios. The present study proposes a multi-scale retrieval experimental framework, encompassing three scale tiers: 100 cattle, 500 cattle, and 658 cattle (full database). The core evaluation metric is Top-K accuracy, with a focus on model performance as database scale expands. The performance retention rate, which has been specially introduced, provides crucial evidence for the quantification of model scalability. This multi-tiered evaluation system reflects current model performance and is capable of predicting future performance trends under expanding data conditions.

As demonstrated in Table 7, upon expanding the database from 100 to 658 subjects, the model’s Top-1 accuracy exhibited a minimal decline of 1.61 percentage points, thereby substantiating its remarkable scalability. This exceptional performance is primarily attributable to the multi-scale feature extraction capability of the EMA attention mechanism, which effectively captures discriminative features across different scales, thereby significantly mitigating feature confusion issues caused by database expansion. It is noteworthy that the Top-10 accuracy maintained an exceptional level above 99.4% across all database scales. This characteristic is of significant practical value in situations where the system is unable to guarantee perfectly accurate first-choice identification. In such cases, the system provides a high-quality candidate list, which significantly narrows the scope of manual review from a full-database search. This enhances the system’s practicality and fault tolerance. Subsequent endeavors will encompass the investigation of the model’s efficacy in the context of ultra-large-scale databases, the perpetual optimization of its recognition capabilities under extreme conditions, and the advancement of the deep application of cattle identification technology within real-world farming environments.

**Table 7:** Model retrieval performance comparison.

Model	Database scale	Top-1 accuracy	Top-10 accuracy	Performance retention rate
CattleMuzzleNet	100 cattle	98.43	99.81	Baseline
	500 cattle	97.14	99.55	98.69
	658 cattle (full dataset)	96.82	99.42	98.36

## 4 Discussion

Despite significant advancements in the realm of deep learning technology for cattle muzzle print recognition, its practical implementation in the domain of livestock insurance remains hindered by challenges in adapting to complex environments. Existing methods are not optimally equipped to handle real-world disturbances, such as image occlusion and angle variations, while also grappling with the trade-off between model accuracy and lightweight requirements. To address this issue, the present study proposes the development of CattleMuzzleNet, a lightweight recognition model integrating Siamese neural networks with EMA attention mechanisms. The employment of a dual-branch weight-sharing architecture ensures precise muzzle pattern matching, thereby providing reliable technical support for precision livestock farming. To validate the system, a large-scale dataset was constructed, comprising 658 cattle and 31,312 images, encompassing multi-angle and occlusion scenarios. A series of comparative experiments with established models such as VGG, MobileNet, and MobileViT was conducted to identify the optimal baseline model. This analysis revealed that MobileViT-S was the optimal model. CattleMuzzleNet was developed on this basis. The experimental findings demonstrate that this model attains 97.87% accuracy and an F1 score of 98.89%, while maintaining lightweight parameters of 6.9 MB. This substantial enhancement in performance when compared to conventional methodologies is indicative of the efficacy of the proposed model. It has been demonstrated that, by optimizing the similarity threshold to 0.5, the model displays excellent robustness in multi-angle and occlusion scenarios. This technology assists farmers in achieving precise herd management and enhances animal welfare by improving individual identification accuracy, thereby comprehensively boosting farming efficiency (47).

However, the study also reveals limitations under certain conditions. The primary issue is the necessity to enhance the accuracy of image recognition in cases where angles are pronounced and occlusion levels are elevated. As demonstrated in Table 6, there is a substantial decline in model performance when the recognition angle exceeds 60 degrees or the occlusion area exceeds 40%. This phenomenon is analogous to the findings observed in cattle face recognition research, suggesting that deep learning methods encounter shared challenges under extreme conditions. It is noteworthy that the recognition of muzzle patterns is more sensitive to occlusion due to its reliance on continuous texture features. In contrast, cattle face recognition can leverage multiple facial landmarks for complementary judgments. Consequently, the dataset proposed in this paper necessitates further expansion. Secondly, the issue of model stability requires further investigation, as images may be affected by noise interference and uneven lighting. Thirdly, the development of keypoint detection algorithms for cattle muzzle patterns is a promising avenue for enhancing recognition performance.

A potential future research direction involves the fusion of features between cattle muzzle ring images and cattle face images. The employment of a mixture-of-experts (MoE) (48) model facilitates the adaptive integration of multimodal information. This fusion strategy has the potential to leverage the complementary strengths of different biometric features: cattle face features demonstrate excellence in frontal recognition, while muzzle-ring features provide reliable texture information during facial occlusion or angular deviation. The MoE framework utilizes a gated network to dynamically evaluate the quality and reliability of each modality’s features, automatically adjusting fusion weights in specific scenarios to enhance system robustness in complex environments. Future research aims to construct a multimodal cattle identification system capable of overcoming current technological limitations, thereby achieving accurate, stable, and real-time identity authentication in complex scenarios.

## 5 Conclusion

This study addresses the practical challenges of muzzle pattern recognition in cattle insurance by proposing the lightweight model CattleMuzzleNet. The dataset under consideration is composed of 31,312 images of cattle belonging to 658 individuals, encompassing the breeds Wagyu, Angus, and Holstein. The dissemination of this dataset will be of considerable benefit to the scientific community, enhancing both the reproducibility and replicability of research findings. The innovation of the selected model lies in the integration of Siamese neural networks, the MobileViT architecture, and the EMA attention mechanism. This approach has been demonstrated to achieve an accuracy of 97.87%, while also maintaining a compact model size of just 6.9 MB. Experimental findings indicate that the similarity threshold of 0.5 achieves optimal performance, achieving a balanced ratio between recognition accuracy and computational efficiency. These achievements signify a pivotal advancement toward the implementation of muzzle-ring pattern recognition technology within embedded systems, thereby establishing a pragmatic foundation for the prevention of insurance fraud and the advancement of digital ranch development.

While the cattle muzzle recognition model performs well under standard conditions, its efficacy in extreme-angle and high-occlusion scenarios highlights the need for future research focused on robustness via strategic data augmentation and multimodal fusion. This work establishes a practical, high-precision, and lightweight framework for biometric identification. By laying the technical groundwork for fraud-resistant embedded systems in agricultural insurance, it contributes significantly to the digital transformation and sustainable development of livestock farming.

## Data Availability

The dataset generated and analysed during this study is publicly available on the Hugging Face Datasets Hub: https://huggingface.co/datasets/MingShuo666/CattleMuzzle.

## References

[1] Ahmad M Abbas S Fatima A Ghazal TM Alharbi M Khan M . Ai-Driven Livestock Identification and Insurance Management System. Egyptian Informatics Journal. (2023) 24:100390. doi: 10.1016/j.eij.2023.100390, 41088955

[2] Hossain ME Kabir MA Zheng L Swain DL McGrath S Medway J. A systematic review of machine learning techniques for cattle identification: datasets, methods and future directions. Artif Intell Agric. (2022) 6:138–55. doi: 10.1016/j.aiia.2022.09.002

[3] Schroeder TC Tonsor GT. International cattle id and traceability: competitive implications for the us. Food Policy. (2012) 37:31–40. doi: 10.1016/j.foodpol.2011.10.005

[4] Kumar S Singh SK. Visual animal biometrics: survey. IET Biom. (2017) 6:139–56. doi: 10.1049/iet-bmt.2016.0017

[5] Barron UG Butler F McDonnell K Ward S The end of the identity crisis? Advances in biometric markers for animal identification. Irish Veterinary Journal (2009)

[6] Awad AI. From classical methods to animal biometrics: a review on cattle identificationand tracking. Comput Electron Agric. (2016) 123:423–35. doi: 10.1016/j.compag.2016.03.014

[7] Neethirajan S Scott S Mancini C Boivin X Strand E. Human-computer interactions with farm animals—enhancing welfare through precision livestock farming and artificial intelligence. Front Vet Sci. (2024) 11:1490851. doi: 10.3389/fvets.2024.1490851, 39611113 PMC11604036

[8] Zhang F Zhao X Wang S Qiu Y Fu S Zhang Y. Research on herd sheep facial recognition based on multi-dimensional feature information fusion Technology in Complex Environment. Front Vet Sci. (2025) 12:1404564. doi: 10.3389/fvets.2025.1404564, 40151568 PMC11948620

[9] Kumar S Tiwari S Singh SK, editors. Face recognition for cattle. 2015 Third International Conference on Image Information Processing (ICIIP); (2015) Waknaghat: IEEE.

[10] Kumar S Tiwari S Singh SK. Face recognition of cattle: can it be done? Proc Natl Acad Sci India Sect A Phys Sci. (2016) 86:137–48. doi: 10.1007/s40010-016-0264-2

[11] Lei X Wen X Li Z. A multi-target cow face detection model in complex scenes. Vis Comput. (2024) 40:9155–76. doi: 10.1007/s00371-024-03301-w

[12] Xiao Z Dai W Li C Liang W Chen X. Enhanced multi-breed cattle face recognition in complex environments using attention-based deep learning. Vis Comput. (2025) 41:9005–24. doi: 10.1007/s00371-025-03910-z

[13] Barron UG Corkery G Barry B Butler F McDonnell K Ward S. Assessment of retinal recognition technology as a biometric method for sheep identification. Comput Electron Agric. (2008) 60:156–66. doi: 10.1016/j.compag.2007.07.010

[14] Rusk CP Blomeke CR Balschweid MA Elliott SJ An evaluation of retinal imaging technology for 4-H beef and sheep identification J Ext 2006 44:9. Available online at: https://archives.joe.org/joe/2006october/a7.php.

[15] Whittier J Doubet J Henrickson D Cobb J Shadduck J Golden B, editors. Biological considerations pertaining to use of the retinal vascular pattern for permanent identification of livestock. Proceedings-American Society of Animal Science Western Section; 2003.

[16] Lowe DG, editor. Object recognition from local scale-invariant features. Proceedings of the seventh IEEE international conference on computer vision; (1999) Kerkyra, Greece: IEEE.

[17] Matera R Angrisani L Neglia G Salzano A Bonavolontà F Verde MT . Reliable use of smart cameras for monitoring biometric parameters in buffalo precision livestock farming. Acta IMEKO. (2023) 12:1–7. doi: 10.21014/actaimeko.v12i4.1638

[18] Kumar S Singh SK Abidi AI Datta D Sangaiah AK. Group sparse representation approach for recognition of cattle on muzzle point images. Int J Parallel Prog. (2018) 46:812–37. doi: 10.1007/s10766-017-0550-x

[19] Awad AI Hassaballah M. Bag-of-visual-words for cattle identification from muzzle print images. Appl Sci. (2019) 9:4914. doi: 10.3390/app9224914

[20] Kusakunniran W Wiratsudakul A Chuachan U Imaromkul T Kanchanapreechakorn S Suksriupatham N . Analysing muzzle pattern images as a biometric for cattle identification. Int J Biom. (2021) 13:367–84. doi: 10.1504/ijbm.2021.117852

[21] Kaur A Kumar M Jindal MK. Shi-Tomasi corner detector for cattle identification from muzzle print image pattern. Ecol Inform. (2022) 68:101549. doi: 10.1016/j.ecoinf.2021.101549

[22] Kumar S Pandey A Satwik KSR Kumar S Singh SK Singh AK . Deep learning framework for recognition of cattle using muzzle point image pattern. Measurement. (2018) 116:1–17. doi: 10.1016/j.measurement.2017.10.064

[23] Bello R-w Talıb AZH Mohamed ASAB. Deep learning-based architectures for recognition of cow using cow nose image pattern. Gazi Univ J Sci. (2020) 33:831–44. doi: 10.35378/gujs.605631

[24] Shojaeipour A Falzon G Kwan P Hadavi N Cowley FC Paul D. Automated muzzle detection and biometric identification via few-shot deep transfer learning of mixed breed cattle. Agronomy. (2021) 11:2365. doi: 10.3390/agronomy11112365

[25] Li G Erickson GE Xiong Y. Individual beef cattle identification using muzzle images and deep learning techniques. Animals. (2022) 12:1453. doi: 10.3390/ani12111453, 35681917 PMC9179917

[26] Kumar S Chaube MK Kumar S. Secure and sustainable framework for cattle recognition using wireless multimedia networks and machine learning techniques. IEEE Trans Sustain Comput. (2021) 7:696–708. doi: 10.1109/TSUSC.2021.3123496

[27] Kimani G Oluwadara P Fashingabo P Busogi M Luhanga E Sowon K . Cattle identification using muzzle images and deep learning techniques. arXiv preprint. (2023) arXiv:231108148.

[28] Lee T Na Y Kim BG Lee S Choi Y. Identification of individual Hanwoo cattle by muzzle pattern images through deep learning. Animals. (2023) 13:2856. doi: 10.3390/ani13182856, 37760256 PMC10525771

[29] Li D Li B Li Q Wang Y Yang M Han M. Cattle identification based on multiple feature decision layer fusion. Sci Rep. (2024) 14:26631. doi: 10.1038/s41598-024-76718-x, 39496678 PMC11535200

[30] Pathak PD Prakash S. Attention-based multi-modal robust cattle identification technique using deep learning. Comput Electron Agric. (2025) 238:110747. doi: 10.1016/j.compag.2025.110747

[31] Russel NS Selvaraj A. Two stream Cnn for muzzle print recognition using Gabor filters. Multimed Tools Appl. (2025) 84:34285–301. doi: 10.1007/s11042-025-20604-9, 41415797

[32] Bara S Das A Singh M Pandey HO Gaur GK Pandey AK . Deep learning assisted muzzle-based identification of Vrindavani cattle–a crossbred of India. J Dairy Res. (2025) 92:167–73. doi: 10.1017/s0022029925101118, 41088955

[33] Xiao Z Yu F Liu L Peng T Hu X Jiang M. Dsanet: a lightweight hybrid network for human action recognition in virtual sports. Comput Anim Virt Worlds. (2024) 35:e2274. doi: 10.1002/cav.2274

[34] Zhang X Xuan C Ma Y Tang Z Cui J Zhang H. High-similarity sheep face recognition method based on a Siamese network with fewer training samples. Comput Electron Agric. (2024) 225:109295. doi: 10.1016/j.compag.2024.109295

[35] Hadsell R Chopra S LeCun Y, editors. Dimensionality reduction by learning an invariant mapping. 2006 IEEE computer society conference on computer vision and pattern recognition (CVPR'06); (2006) New York, NY: IEEE.

[36] Wen Y Luo B Shi W Ji J Cao W Yang X . Sat-net: structure-aware transformer-based attention fusion network for low-quality retinal fundus images enhancement. IEEE Trans Multimedia. (2025) 27:6198–6210. doi: 10.1109/TMM.2025.3565935

[37] Cochran WG. Relative accuracy of systematic and stratified random samples for a certain class of populations. Ann Math Stat. (1946) 17:164–77. doi: 10.1214/aoms/1177730978

[38] Xiao Z Chen Y Zhou X He M Liu L Yu F . Human action recognition in immersive virtual reality based on multi-scale spatio-temporal attention network. Comput Anim Virt Worlds. (2024) 35:e2293. doi: 10.1002/cav.2293

[39] Shen P Sun N Hu K Ye X Wang P Xia Q . Firevit: an adaptive lightweight backbone network for fire detection. Forests. (2023) 14:2158. doi: 10.3390/f14112158

[40] Pan F Zhang B Zhao X Shuai L Chen P Duan X. A lightweight, secure authentication model for the smart agricultural internet of things. Agronomy. (2023) 13:2257. doi: 10.3390/agronomy13092257

[41] Chen Z Zhou H Lin H Bai D. Teavitnet: tea disease and pest detection model based on fused multiscale attention. Agronomy. (2024) 14:633. doi: 10.3390/agronomy14030633

[42] Ding Y Huang H Cui H Wang X Zhao Y. A non-destructive method for identification of tea plant cultivars based on deep learning. Forests. (2023) 14:728. doi: 10.3390/f14040728

[43] Lin X Sun S Huang W Sheng B Li P Feng DD. Eapt: efficient attention pyramid transformer for image processing. IEEE Trans Multimed. (2021) 25:50–61. doi: 10.1109/TMM.2021.3120873, 41116384

[44] Zhao Y Zhang H Lu P Li P Wu E Sheng B. Dsd-matchingnet: deformable sparse-to-dense feature matching for learning accurate correspondences. Virtual Real Intell Hardw. (2022) 4:432–43. doi: 10.1016/j.vrih.2022.08.007

[45] Petersen F Kuehne H Borgelt C Deussen O, editors. Differentiable top-K classification learning. International Conference on Machine Learning; (2022) Baltimore, Maryland: PMLR.

[46] Ruchay A Kolpakov V Guo H Pezzuolo A. On-barn cattle facial recognition using deep transfer learning and data augmentation. Comput Electron Agric. (2024) 225:109306. doi: 10.1016/j.compag.2024.109306

[47] Angrisani L Amato A Amato F Autiello MA Bonavolontà F Matera R ., editors. Buff4l. 0: veterinary and engineering sciences at the crossroads in the industry 4.0 age. 2021 IEEE 6th international forum on research and Technology for Society and Industry (RTSI); (2021) Naples, Italy: IEEE.

[48] Chen Z Deng Y Wu Y Gu Q Li Y. Towards understanding the mixture-of-experts layer in deep learning. Adv Neural Inf Proces Syst. (2022) 35:23049–62. doi: 10.48550/arXiv.2208.02813

[49] Barry B Gonzales-Barron U McDonnell K Butler F Ward S. Using muzzle pattern recognition as a biometric approach for cattle identification. Trans ASABE. (2007) 50:1073–80. doi: 10.13031/2013.23121

[50] Ouyang D He Zhang G . Efficient multi-scale attention module with cross-spatial learning[C]//ICASSP 2023-2023 IEEE international conference on acoustics, speech and signal processing (ICASSP). IEEE, (2023): 1–5.

